# Validation of single-step genomic predictions using the linear regression method for milk yield and heat tolerance in a Thai-Holstein population

**DOI:** 10.14202/vetworld.2021.3119-3125

**Published:** 2021-12-15

**Authors:** Piriyaporn Sungkhapreecha, Ignacy Misztal, Jorge Hidalgo, Daniela Lourenco, Sayan Buaban, Vibuntita Chankitisakul, Wuttigrai Boonkum

**Affiliations:** 1 Department of Animal Science, Faculty of Agriculture, Khon Kaen University, Thailand; 2 Department of Animal and Dairy Science, University of Georgia, USA; 3 The Bureau of Animal Husbandry and Genetic Improvement, Pathum Thani, Thailand; 4 Network Center for Animal Breeding and Omics Research, Khon Kaen University, Thailand

**Keywords:** accuracy, genomic selection, heat stress, linear regression method, ssGBLUP

## Abstract

**Background and Aim:**

Genomic selection improves accuracy and decreases the generation interval, increasing the selection response. This study was conducted to assess the benefits of using single-step genomic best linear unbiased prediction (ssGBLUP) for genomic evaluations of milk yield and heat tolerance in Thai-Holstein cows and to test the value of old phenotypic data to maintain the accuracy of predictions.

**Materials and Methods:**

The dataset included 104,150 milk yield records collected from 1999 to 2018 from 15,380 cows. The pedigree contained 33,799 animals born between 1944 and 2016, of which 882 were genotyped. Analyses were performed with and without genomic information using ssGBLUP and BLUP, respectively. Statistics for bias, dispersion, the ratio of accuracies, and the accuracy of estimated breeding values were calculated using the linear regression (LR) method. A partial dataset excluded the phenotypes of the last generation, and 66 bulls were identified as validation individuals.

**Results:**

Bias was considerable for BLUP (0.44) but negligible (−0.04) for ssGBLUP; dispersion was similar for both techniques (0.84 vs. 1.06 for BLUP and ssGBLUP, respectively). The ratio of accuracies was 0.33 for BLUP and 0.97 for ssGBLUP, indicating more stable predictions for ssGBLUP. The accuracy of predictions was 0.18 for BLUP and 0.36 for ssGBLUP. Excluding the first 10 years of phenotypic data (i.e., 1999-2008) decreased the accuracy to 0.09 for BLUP and 0.32 for ssGBLUP. Genomic information doubled the accuracy and increased the persistence of genomic estimated breeding values when old phenotypes were removed.

**Conclusion:**

The LR method is useful for estimating accuracies and bias in complex models. When the population size is small, old data are useful, and even a small amount of genomic information can substantially improve the accuracy. The effect of heat stress on first parity milk yield is small.

## Introduction

The development of a sustainable animal production system in tropical countries in the face of climate change is challenging. Heat stress is an important concern in dairy production systems in places that are always hot and humid, such as Thailand [1]. The effects of heat stress on animals include decreased production and fertility, as well as health issues. Heat stress can be addressed by managing the animals’ housing and nutrition [2-7]. However, this requires expensive technology and is both temporary and not sustainable. The genetic component of heat stress can be estimated using a random regression animal model with the temperature-humidity index (THI) as a covariable [8]. Genetic selection for heat tolerance is cumulative and permanent and represents a powerful tool that could be used to improve the performance and fitness of dairy cattle [1]. The selection of heat-tolerant animals would be a cheap input technology that could play a key role in lessening the harmful impacts of abiotic factors on agricultural production [9,10].

Genomic selection relies on genomic estimated breeding values (GEBV) based on dense single nucleotide polymorphism (SNP) information. Genomics increases the genetic gain through more accurate breeding values and by reducing the generation interval [11], especially for young dairy bulls with no daughter records. Genomic selection can therefore significantly reduce economic costs compared with the traditional progeny testing schemes. Single-step genomic best linear unbiased prediction (ssGBLUP) has become the method of choice for genomic evaluations [12] because it combines all pedigree information, genotypes, and phenotypes to compute GEBV, providing more accurate predictions [13-15]. To assess the accuracy of predictions, Legarra and Reverter [16] proposed the linear regression (LR) method, which relies on the comparison of (G)EBV obtained from a partial dataset in which validation bulls do not have daughters with phenotypic records, with (G)EBV predicted from a whole dataset (i.e., including phenotypic records of daughters of validation bulls). These authors used cross-validation to compare and validate genomic predictions using statistical measures of bias, dispersion, and accuracy. The LR method is easy to use, robust, and suitable, especially for small datasets [17]. The value of data from distant generations on the accuracy of predictions for selection candidates can be small. Lourenco *et al*. [18] reported that the accuracy of genomic evaluations for the final score in U.S. Holsteins did not decrease when excluding data from two or three more distant generations (12-17 years of data), and there was a reduction in computing cost. We hypothesized that the use of genomic information improves bias, dispersion, and the accuracy of estimated breeding values in the Thai-Holstein population and that using the most recent 10 years of data is enough to maintain the accuracy of predictions.

This study was conducted to assess the benefits of using ssGBLUP for genomic evaluations of milk yield and heat tolerance in Thai-Holstein cows and to test the value of old phenotypic data to maintain the accuracy of predictions.

## Materials and Methods

### Ethical approval

Animal welfare and use committee approval was not needed for this study as datasets were obtained from pre-existing databases based on routine animal recording procedures.

### Study period and location

The study was conducted from September 2020 to March 2021. The study was carried at the Laboratory of Animal Breeding and Genetics, Department of Animal and Dairy Science, College of Agricultural and Environmental Sciences, University of Georgia.

### Data

The dataset after editing included 104,150 test-day milk yield records from the first parity of 15,380 Thai-Holstein cows recorded from 1999 to 2018. The Thai-Holstein is a tropical crossbreed with 75% or more Holstein genetics from the United States and Canada, and 25% or less Thai Native or Brahman cattle. The Thai-Holstein breed has high fertility, the ability to adapt to hot and humid conditions, and is resistant to parasites and diseases. Test-day milk yield records were obtained from the Bureau of Biotechnology in Livestock Production of the Department of Livestock Development and were collected as part of the Master Bull project implemented from 1999 to 2018. Quality control on phenotypic data was performed to exclude: (1) records taken before 6 days in milk or after 305 days in milk; (2) herd-test-date combinations that had records coming from <30 cows or daughters of less than three sires; and (3) records from herds that used less than three sires and from daughters of sires that were used in less than three herds. Only cows with at least 5 test day records during lactation were used in the analyses.

The pedigree contained 33,799 animals born between 1944 and 2016, among which 882 animals genotyped using the Illumina BovineSNP50 Bead Chip (Illumina Inc., San Diego, CA) were analyzed. Quality control of genomic data retained SNP and animals with call rates >0.9 and SNP with minor allele frequencies >0.05. All the animals and a total of 43,288 SNP markers were retained for further analyses. The number of animals with pedigree, genotypic, and phenotypic information is presented in Table-1.

**Table 1:** Data structure and descriptive statistics for milk yield in the full dataset (1999-2018) or the last 10 years of data (2009-2018) of the Thai-Holstein population.

Item/years of data	1999-2018	2009-2018
Number of contemporary groups		
Herd×Month×Year	24,928	13,074
Farm×Season	218	125
Breed group×Day in milk	30	30
Classes of ages at first calving	7	7
Number of breed groups	3	3
Number of animals with records	15,380	8,290
Number of animals with pedigree	33,799	21,212
Number of animals with genotype	882	882
Descriptive statistics of milk yield		
Minimum (kg)	5.0	5.0
Maximum (kg)	45.0	44.0
Mean (kg)	14.0	14.4
SD (kg)	4.5	4.5
Number of records	104,150	58,905

SD=Standard deviation

Climate data were obtained from weather stations from the Thai Meteorological Department in all regions of Thailand closest to each dairy farm based on postal code (no longer than 20 km). These data included daily temperature and relative humidity recorded every 3 h, which were used to calculate the THI based on the formula used by the National Oceanic and Atmospheric Administration [19] as follows:

THI=(1.8T+32)–(0.55–0.0055RH)(1.8T–26),

in which T is the temperature in degrees Celsius and RH is the relative humidity as a percentage. The mean daily THI for each milk test date was used to detect the threshold of heat stress and to estimate genetic parameters as suggested by Bohmanova *et al*. [20].

### Analyses and computations

Genetic parameters and breeding values were estimated using the REMLF90 program [21] for a repeatability model with random regressions on a THI function as follows:

y_ijklmn_=hmy_i_+fs_j_+bgdim_k_+afc_l_+bg_m_[f(THI)_i_]+a_n_+a_htn_ [f(THI)_i_]+p_n_+p_htn_ [f(THI)_i_]+e_ijklmn_,

in which y_ijklmn_ is the test-day milk yield of cow n; hmy_i_ is the random effect of the i^th^ combination of herd-month-year of the test (i=1-24,928); fs_j_ is the fixed effect of the j^th^ combination of farm and calving season (j=1-218); bgdim_k_ is the fixed effect of the k^th^ combination of the breed group and the days in milk group (k=1-30); afc_l_ is the fixed effect of the l^th^ class of age at first calving (l=1-7); bg_m_ is a fixed effect of the m^th^ breed group (m=1-3); a is a general random additive genetic effect (without considering heat tolerance); a_ht_ is the random additive genetic effect for heat tolerance; p is a general random permanent environmental effect (without considering heat stress); p_ht_ is the random permanent environmental effect of heat tolerance; e is the random residual effect; and f(THI) is a function of the THI defined as follows:

f(THI)=


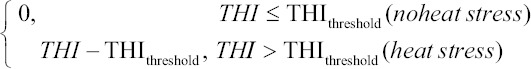


The repeatability model with random regressions on the THI function captures the effects of heat stress on milk yield because it models milk yield as a function of degrees of heat stress (reaction norm approach) affecting the animals. The intercept additive genetic effect, which is independent of the THI function and represents the intercept, indicates the animal’s capacity to produce milk under thermoneutral conditions. The additive genetic effect regressed on the THI function quantifies the animal’s sensitivity to heat stress; it represents the slope of the reduction in milk yield per THI unit increase above the threshold of thermoneutral conditions [22]. To define the threshold, analysis with the full dataset by ssGBLUP was undertaken by varying the THI values from 72 to 80. The THI level in the analysis with the lowest −2logL value was selected as the threshold (data not shown).

The (co)variance structure among all random effects in the model was:


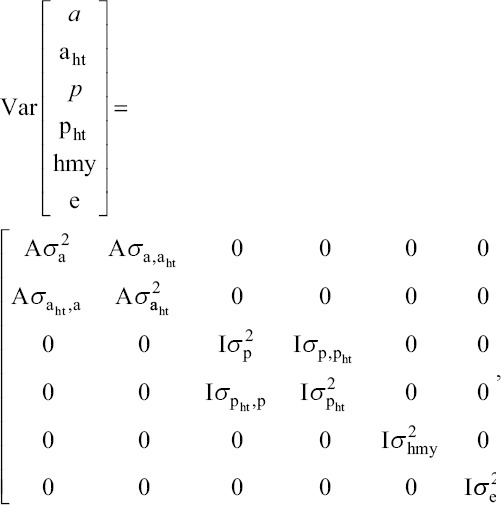


in which A is the numerator relationship matrix; I is the identity matrix; σ^2^ is the variance; σ is the covariance; and a, a_ht_, p, and p_ht_ are defined as above.

Analyses were performed with and without genomic information using ssGBLUP and Best linear unbiased prediction (BLUP), respectively. In ssGBLUP, the inverse of the pedigree relationship matrix (A) in the mixed model equations is replaced by the inverse of the realized relationship matrix (H) [13], which is:


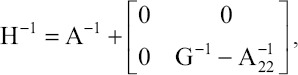


In which A^-1^_22_ is the inverse of the pedigree relationship matrix for genotyped animals only and is G^-1^ the inverse of the genomic relationship matrix (G), constructed as in VanRaden [23] using current allele frequencies. To avoid singularity problems, G was blended with 5% of A_22_, the elements for genotyped animals in A. The method to analyze scaling to make G compatible with A followed by Chen *et al*. [24].

Estimated breeding values were calculated following [8]:

BV=a+f(THI)*a_ht_

in which is the regular breeding value, is the heat-tolerance breeding value, and f(THI) is defined as above. The effects of heat tolerance determine the change in breeding values of animals per THI unit increase. For example, if a_ht_ of one animal is −0.1 kg, and it is 0.0 kg for a second animal, the second animal has a superiority of 0.1 kg at f(THI)=1 and of 0.5 at f(THI)=5, assuming that is equal for both animals.

### Validation of predictions

Statistics from the LR method [16] were used to validate breeding values with and without accounting for heat tolerance. A partial dataset (p) was constructed by removing the phenotypes of the last generation (6 years). The validation group consisted of 66 bulls born between 1996 and 2011 that did not have daughters with milk yield records in the partial dataset but had an average of 4 (ranging from 1 to 23) daughters with records in the whole dataset (w). Validation was done assuming predictions were calculated for a thermo-neutral environment with a THI of 76 and an extreme environment with a THI of 80.

Statistics for bias, dispersion, the ratio of accuracies, and accuracies were calculated as follows.

Bias was computed as:


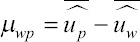


in which 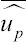 and 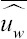 are the mean estimated breeding values obtained with the partial and whole datasets, respectively; μ_wp_ has an expected value of 0 if the evaluation is unbiased.

The slope or dispersion of the regression of 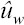 on 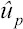 is expected to be 1 if there is no over/under dispersion. It was computed as:


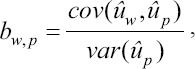


In which 
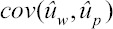
 is the covariance between breeding values obtained with the whole and partial datasets, and 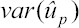 is the variance of estimated breeding values obtained with the partial dataset.

An estimator of the ratio of accuracies was calculated as the correlation between 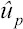 and 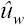 as follows:


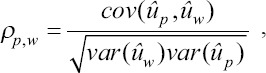


The ratio of accuracies is an estimator of the consistency between subsequent evaluations, and a greater value indicates more stable predictions.

The accuracy of genomic predictions was calculated as follows:


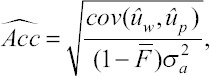


in which is the average inbreeding coefficient in the individuals in the validation group, and is the additive genetic variance.

## Results and Discussion

The threshold for heat stress in this population was estimated to be a THI of 76, which means that milk yield began to decrease at this THI level. To define the threshold, analysis with the full dataset was undertaken by varying the THI values from 72 to 80. The THI level in the analysis with the lowest −2logL value was selected as the threshold (data not shown; supplementary data is available from https://doi.org/10.3168/jds.2021-20151). The estimate for intercept additive genetic variance at a THI of 76 was 4.89±0.03 kg^2^, and for the slope genetic variance of heat tolerance, it was 0.004±0.002 kg^2^. Therefore, considerably low genetic variation was found for heat tolerance. This is possibly because the Thai-Holstein population is composed of crossbred animals, and the local breeds that are crossed with Holstein are well-adapted to hot environments. In addition, this population has been under selection for heat tolerance since 1992. Aguilar *et al*. [25] found that genetic variance for heat tolerance doubled from first to second parity and increased by ~20% from second to third parity. Because only first parity records were available in our study, future research is needed to evaluate the genetic variation for heat tolerance on later parities.

Statistics for bias, dispersion, the ratio of accuracies, and accuracy are presented in Table-2. Bias was considerable (0.44) for BLUP evaluations but was marginal (−0.04) for ssGBLUP evaluations. For a THI of 76, dispersion was 0.84 for BLUP and 1.06 for ssGBLUP, indicating inflation of EBV estimates, whereas GEBV were only marginally deflated. Tsuruta *et al*. [26] suggested that values of dispersion close to 1 indicate that early evaluations are successful in predicting the actual magnitude of differences among animals and stated that deviations of ±15% from 1 are in general acceptable. The ssGBLUP in our study yielded estimates within this range.

**Table 2:** Statistics from the LR validation of predictions obtained with traditional BLUP and ssGBLUP for a THI of 76 and 80 using data from 1999 to 2018 or the past 10 years of data (2009-2018).

Years of data	1999-2018		2009-2018	
		
Method	BLUP	ssGBLUP	BLUP	ssGBLUP
LR statistics				
THI of 76				
Bias	0.44	−0.04	0.54	-0.10
Slope or dispersion	0.84	1.06	0.75	1.00
Ratio of accuracies	0.33	0.97	0.13	0.95
Accuracy	0.18	0.36	0.09	0.32
−2logL	483,163.91	483,145.90	275,019.18	275,004.30
AIC	483,179.91	483,161.90	275,035.18	275,020.30
THI of 80				
Bias	0.36	−0.04	0.45	-0.10
Slope or dispersion	0.89	1.07	0.66	1.01
Ratio of accuracies	0.34	0.97	0.12	0.96
Accuracy	0.18	0.34	0.08	0.30
−2logL	485,919.19	485,920.17	276,395.05	276,398.67
AIC	485,935.19	485,936.17	276,411.05	276,414.67

AIC=Akaike information criterion, BLUP=Best linear unbiased prediction, LR=Linear regression; ssGBLUP=Single-step genomic best linear unbiased prediction, THI=Temperature-humidity index

The ratio of accuracies is a metric of the stability of genetic evaluations. It can also be interpreted as the inverse of the relative gain in accuracy due to the addition of new phenotypes [27]. As expected, the statistic for the ratio of accuracies was lower in BLUP (0.33) evaluations than in ssGBLUP (0.97) evaluations, indicating that ssGBLUP evaluations were more stable and that GEBV of validation bulls obtained without daughter records were estimated more accurately. These results are in agreement with those reported by Macedo *et al*. [28] in a research study with a dairy sheep population.

In general, the LR statistics deteriorated for a THI of 80, especially the bias from BLUP predictions. This is possible because of the low genetic variance found for heat tolerance. Combining genomic information with the data helped to keep bias, dispersion, the ratio of accuracies, and accuracy at more similar levels under a THI of 76 and 80. Fragomeni *et al*. [29] showed that the accuracy of GEBV was superior to the accuracy of estimated breeding values (EBV), even in extreme environments, and concluded that genomic information helps to better identify animals that perform well in a range of environments. Having highly accurate GEBV given an extreme THI is important when production levels decrease as a result of heat stress. The impact of heat stress in this Thai-Holstein population was small, probably because: (1) The housing conditions are proper, so the external THI does not reflect the conditions inside the barns; (2) only data for the first parity were available; and (3) the population is composed of crossbred animals, in which Holsteins are crossed with adapted, local breeds.

The accuracy of predictions is a crucial component in animal breeding programs because it has a direct relationship with the selection response. The accuracy of ssGBLUP predictions doubled compared with those from BLUP, which has the potential to increase the selection response by two-fold. The gain in accuracy due to the use of genomic information is explained by more accurate estimates of the Mendelian sampling terms [30]. Macedo *et al*. [28] reported an increased accuracy (~33%) for milk yield predictions in ssGBLUP compared with BLUP in a dairy sheep population. Cesarani *et al*. [31] showed a gain of ~37% in the accuracy for milk ability predictions in dual-purpose cattle when comparing ssGBLUP and BLUP.

There is a question as to why the accuracy doubled despite having only a few genotyped animals. First, the accuracies are relatively small because the population size is small. The genomic improvement with genomic relationships is due to the estimation of clusters of chromosome segments [32]. With few sires, the number of such clusters could be relatively small, and genomic predictions could be accurate within the population but not across populations. Schultz and Weigel [33] found that the accuracies of prediction based on reference from one herd applied to that herd could be higher than if the reference population includes several herds.

Another possible factor to explain the two-fold increase in the accuracy of predictions is related to the identification of “hidden relationships” in the pedigree. The percentage of animals with both parents missing was 23.1%, and with one parent missing, it was 7.2%. Therefore, animals unrelated through pedigree relationships may become related in the genomic relationship matrix, contributing to a more precise estimation of the effects of independent chromosome segments. Unknown parent groups are widely used in the modeling of missing pedigrees. In this method, breeding values of animals with missing pedigrees are adjusted according to the fraction of genes originating from each unknown parent group [34]. The accurate estimates of unknown parent groups rely on having many observations [35,36]. In this research study, the small size of the dataset limited our ability to investigate the differences in the accuracy of predictions with and without unknown parent groups in the model. This topic deserves future research.

The statistics for bias, dispersion, the ratio of accuracies, and accuracy (Table-2) in general deteriorated when removing the first 10 years of phenotypic data compared with using the full dataset. The deterioration was more evident for BLUP evaluations than for ssGBLUP evaluations. For example, accuracy dropped from 0.18 to 0.09 in BLUP and from 0.36 to 0.32 in ssGBLUP evaluations when assuming a THI of 76. A similar drop was observed for a THI of 80. Although there was a drop in predictions from ssGBLUP, the drop was less than from BLUP. Therefore, genomic evaluations were more persistent when older data were removed. The difference in computing time was negligible when using phenotypes from 1999 to 2008 or 2009 to 2018 (data not shown). Although the deterioration was not dramatic for ssGBLUP evaluations, our recommendation is to use all the data available in the breeding program to maximize accuracy and genetic progress, given the small size of this population. In large populations, the contribution of old data for predicting future breeding values of selection candidates is negligible [18,31].

**Figure-1:**
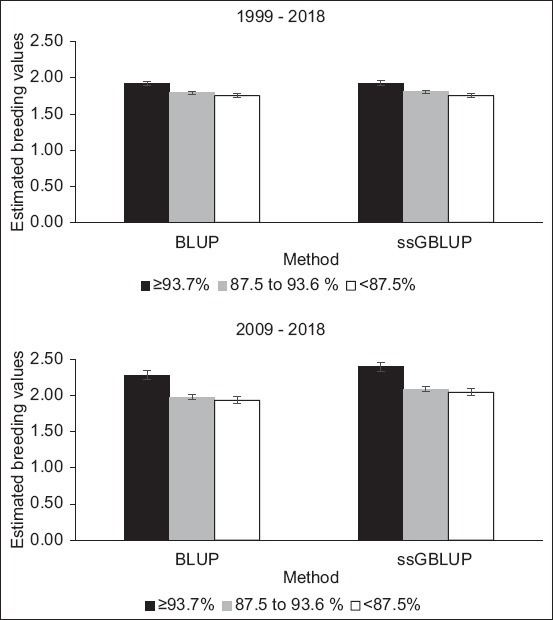
The different estimated breeding values and genomic estimated breeding values for milk yield and heat tolerance traits by breed groups. The analysis considered the top 20% of the herd using the BLUP and ssGBLUP methods. BLUP=Best linear unbiased prediction, ssGBLUP=Single-step genomic best linear unbiased prediction.

Figure-1 shows the different EBV and GEBV estimations for milk yield and heat tolerance traits when analyzed from the top 20% of the herd. The GEBV was apparently higher than EBV in all breed groups. These findings indicate that the rates of genetic progress for milk and heat tolerance when using the ssGBLUP method are faster than when using the BLUP method, a factor that is meaningful for decreasing the generation interval, increasing the selection accuracy, and reducing the costs of progeny testing. However, the number of individuals in the reference population related to the data set and the number of SNPs are mainly considered factors for better accuracy of this method in genetic evaluation of dairy breeding programs.

## Conclusion

Genomic selection could be used to improve milk yield production in the Thai-Holstein population with the potential to double genetic gain. It is important to make use of all the available information to maximize the accuracy of genomic predictions. The accuracy of breeding values for thermo-neutral and extreme environments were similar mainly because marginal genetic variation was found for heat tolerance and a small reference population dataset was used. It is important to highlight the necessity of future research, including data from more parity to explore the potential of genetic progress for heat tolerance.

## Authors’ Contributions

PS: Analyzed the data. PS, JH, DL, IM, and WB: Drafted the manuscript. PS, JH, IM, and DL: Reviewed and edited the manuscript. IM, PS, JH, and DL: Contributed to conceptualization. SB: Analyzed the samples. WB and VC: Supervised the research. All authors read and approved the final manuscript.
